# A unique case report of mulberry second molar in a non-syphilitic patient

**DOI:** 10.1097/MD.0000000000039127

**Published:** 2024-07-26

**Authors:** Zdravka Harizanova, Ferihan Popova, Marieta Peycheva

**Affiliations:** a Department of Anatomy, Histology and Embryology, Faculty of Medicine, Medical University-Plovdiv, Plovdiv, Bulgaria; b Department of Neurology, Faculty of Medicine, Medical University-Plovdiv, Plovdiv, Bulgaria; c Research Institute, Faculty of Medicine, Medical University-Plovdiv, Plovdiv, Bulgaria.

**Keywords:** congenital syphilis, developmental enamel diseases, Hutchinson triad, mulberry molars

## Abstract

**Rationale:**

The common embryologic neural origin of the ectoderm includes the epidermal layer of the skin and the amelodentinal components of the teeth which can result in numerous diseases damaging both skin and dentition. Three main dental abnormalities were described as a consequence of congenital syphilis: Hutchinson incisors, bud molars, and mulberry molars which usually affect all permanent first molars.

**Patient concerns:**

As far as we know, this case is the first reported for mulberry second molar in a non-syphilitic patient. Ten projections globular in shape were presented on the occlusal surface of the second mandibular molar.

**Diagnoses:**

The findings were characteristic for mulberry molars and therefore diagnosed as mulberry molars.

**Interventions and outcomes:**

No prosthetic treatment was conducted, just topical fluoride application was performed, and periodic observations were scheduled.

**Lessons:**

It is recommended that gynecologists and pediatricians should be provided with additional information about the local and systemic factors that can lead to developmental diseases of the teeth.

## 1. Introduction

The final size, shape, and structure of a tooth is the result of the finely coordinated onset and rate of cellular proliferation within the tooth germ, and the onset, rate, and duration of mineralization during dentinogenesis and amelogenesis. Amelogenesis is the process of formation of the enamel by the ameloblasts and it has 3 phases: (a) phase of production of enamel organic matrix; (b) phase of mineralization of the matrix; (c) phase of enamel maturation. Any dysfunctions of the ameloblasts caused by different environmental factors lead to various defects of the enamel. These factors can be local and systemic. Local factors such as inflammation of the tooth bud usually cause local defects, and they vary depending on the period during which these factors act. If they act during the late enamel maturation the result will be nontransparent opaque enamel, if the period is early maturation the result will be hypoplastic enamel defects.^[1]^ Systemic factors are birth trauma, drugs, prolonged illness of the mother, and metabolic disorders.^[2]^ Infections such as congenital syphilis can destroy the ameloblasts and odontoblasts which will lead to developmental defects of enamel or dentin. They can vary from dental color, shape, or size alteration to total enamel absence. The triad of Hutchinson teeth (peg-shaped maxillary permanent incisors), interstitial keratitis and hearing loss is pathognomonic of congenital syphilis.^[3]^ Other signs can be mulberry teeth which are defective first molars with additional globular-shaped cusps. The infection affects these teeth probably because the first permanent tooth where calcification originates is the first molar and the process occurs at the time of birth, maxillary incisors being the second. This is also the first tooth that erupts in the oral cavity which takes place at around 6 years of age.^[4]^

We believe that this is a unique case with the appearance of a second permanent molar resembling mulberry molar in a non-syphilitic patient.

## 2. Case report

**Figure 1.**
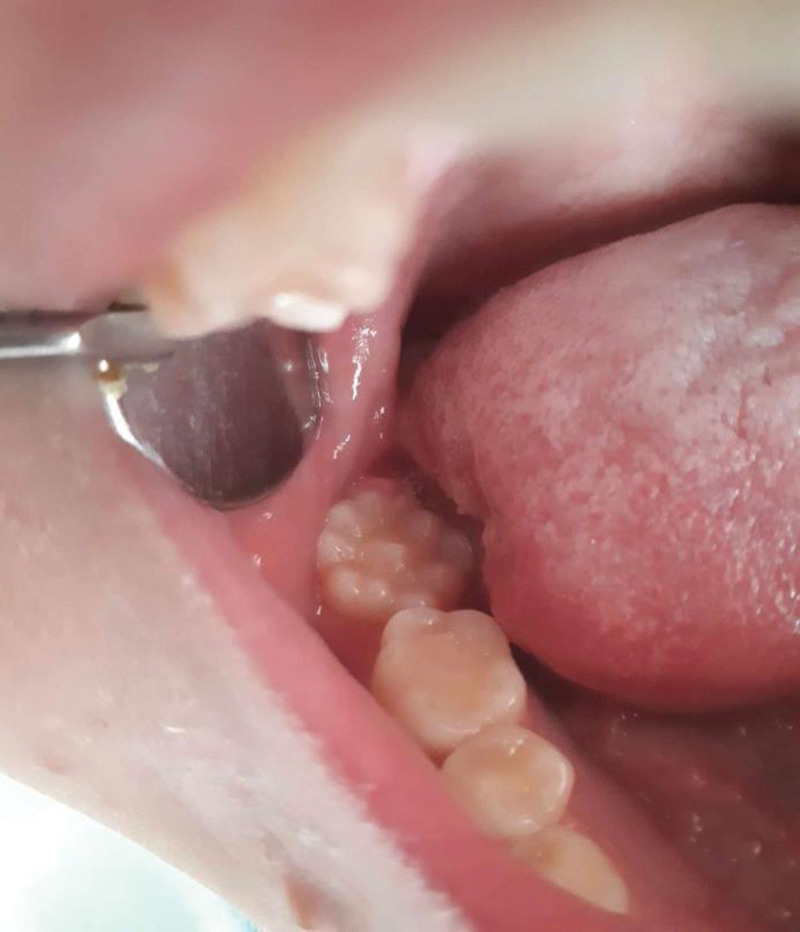
Mulberry second mandibular left molar.

An 18-year-old girl visited dental clinic in Varna for medical dental check. Extraoral examination exhibited no peculiarities such as lymphadenopathy. Oral examination revealed no gingival inflammatory process, no calculus, and no soft-tissues pathology. Numerous additional cusps were detected on the occlusal surface of the mandibular right second molar. The projections were globular in shape, 1 in the center and 9 in the periphery of the surface, 2 mm in size. The tooth resembled mulberry molar which is characteristic of congenital syphilis. On the following clinical examination, no additional cusps were found neither on the occlusal surface of the first molars, nor on the surface of the rest of the second molars. The maxillary antagonists occluded well with the tooth and the occlusion was orthognathic. No caries was detected in the fissures between the cusps. The medical and personal history of the patient was noncontributory. The parents and the relatives did not exhibit such abnormalities. The mother had normal pregnancy without diseases or fever, or any history of syphilis (Fig. 1).

Further investigations were performed. Conventional blood test revealed no abnormalities, normal hemoglobin %, erythrocytes, differential leukocyte count, and platelet count. Screening test for venereal diseases was normal and specific test for *Treponema pallidum* was negative.

Since the patient had no complaints about the occlusion or the function of the tooth, no prosthetic treatment was conducted. The additional cusps were treated with fluoride.

An ethical approval was taken for this publication by the Ethics committee in Medical University-Plovdiv. Informed consent was taken from the patient.

## 3. Discussion

In this case abnormal occlusal surface of the second mandibular molar was detected with the presence of numerous additional cusps globular in shape which were not found either on the first molars, nor on the rest of the second molars. These peculiarities are characteristic for congenital syphilis and therefore diagnosed as mulberry molars.

The common embryological neural origin of the ectoderm involves the epidermal layer of the skin and the amelodentinal components of the teeth that can result in numerous conditions affecting both skin and dentition.^[3]^ Congenital syphilis arises from transplacental fetal infection with *T pallidum* during pregnancy from an untreated mother. *T pallidum* crosses the placenta after the 16th week of intrauterine life, and it damages the facial and dental structures of the embryo.^[5]^ Manifestations can be early which are mucocutaneous lesions on the lips, tongue, skin, and palate. Late congenital syphilis manifests with abnormalities of the eyes: optic atrophy, the ears: deafness, the bones: short maxilla, saddle nose, palatal perforation, the central nervous system: mental diseases, paralysis, and paresis.

Sir Jonathan Hutchinson, Assistant Surgeon at the London Hospital in 1861 was the first who described dental manifestations of congenital syphilis. Congenital syphilis affects the amelogenesis of the molars and incisors and the 3 main dental defects are Hutchinson incisors, Moon molars (bud molars), and Fournier molars (mulberry molars).^[6]^ Putkonen (1962) examined 235 patients with syphilis and found 45% of them were with Hutchinson incisors and 22% of them were with Moon molars.^[7]^ Hutchinson described the incisors as short and narrow, screwdriver shaped with a deep vertical notch.^[8]^ Henry Moon first described small and dome shaped first molars with wide base and narrow at the cusps, smooth surface, and no grooves.^[9]^ Later these teeth were termed as Moon molars or bud molars. Fournier described permanent first molar defect in which there is a groove around each of the cusps which resemble a small tooth growing out of larger one and probably results from infection at a slightly different time of development.^[10]^ These molars are dwarfed by small occlusal surface and roughened lobulated hypoplastic enamel leading to caries.^[11]^ The surface has additional poorly formed cusps, narrow at the grinding surface than at its base. They are referred to as mulberry molars, they are normal in the cervical and middle third, the defect is considered to be enamel hypoplasia which exposed the molars to increased risk of caries because of uneven enamel formation.

Massler and Schour investigated teeth with congenital syphilis and postulated that only teeth developing during neonatal period can be affected. They reported that these teeth are permanent incisors and first molars which are in morphodifferentiation stage whereas permanent second and third molars which are still in bud stage are usually not affected at all.^[12]^ On the other hand, Butler (1939) and Dahlberg (1945) believed that in each dental arch there is a concentration of morphological genes, the result of which is that each tooth resembles its neighbor. This concentration is highest when the first tooth of each morphological group is developing, so the medial tooth of each group is the most stable and is termed the key tooth. With moving away from the key tooth, this concentration decreases for the more distally positioned tooth. Accordingly, it might not be unreasonable to expect that the later forming teeth would be more likely to be distorted than the earlier forming ones.^[13,14]^

This might explain the fact that in the present study the defected tooth was not the first molar, but the second. Furthermore, not all 4 of them were abnormal, but only the mandibular right one. Defects on a single tooth reveal local etiological factors such as trauma or infection of the tooth bud. Systemic factors affect all the teeth developing during the time of the factor, while defects caused by genetic factors are generalized in distribution. The other interesting fact in the present case is that there was no caries detected on the occlusal surface.

A rare case of no syphilitic mulberry first molars was described in dental clinic in Raichur, but it involves all the first molars.^[15]^ A case of 15-year-old girl was reported by Sedano et al, where the defected teeth were again the first permanent molars.^[4]^ Rodrigo et al reported a case of Hutchinson teeth in a 31-year-old woman which history did not reveal any clues to syphilis and the *T pallidum* particle agglutination test was negative in both the patient and her mother.^[16]^

Therefore, we believe that the cause for this molar anomaly was enamel hypoplasia or a deficiency in dental enamel due to local environmental factor which has acted during the development of the concrete tooth. The deformity was not severe, no esthetic issues were presented and there was no dysfunction of the tooth, no obstruction or caries seen. Hence, no prosthetic treatment was conducted, just topical fluoride application was performed, and periodic observations were scheduled.

## 4. Conclusion

This case is unique because it exhibits mulberry molars which are characteristic for congenital syphilis and affect all first molars in a non-syphilitic patient and affecting of only 1 second molar. Hence, it is recommended that gynecologists and pediatricians should be provided with additional information about the local and systemic factors that can lead to developmental diseases of the teeth.

## Author contributions

**Conceptualization:** Zdravka Harizanova, Ferihan Popova, Marieta Peycheva.

**Formal analysis:** Zdravka Harizanova.

**Methodology:** Zdravka Harizanova, Ferihan Popova.

**Writing – original draft:** Zdravka Harizanova, Ferihan Popova, Marieta Peycheva.

**Supervision:** Ferihan Popova, Marieta Peycheva.

## References

[1] Seow WK Shepherd RW Ong TH. Oral changes associated with end-stage liver disease and liver transplantation: implications for dental management. ASDC J Dent Child. 1991;58:474–80. 1838379

[2] Schour I. The neonatal line in the enamel and dentin of the human deciduous teeth and first permanent molar. J Am Dent Assoc. 1936;23:1946–55. 10.1177/00220345460250030601 20988348

[3] Freiman A Borsuk D Barankin B Sperber GH Krafchik B. “Dental manifestations of dermatologic conditions.”. J Am Acad Dermatol. 2009;60:289–98. 19027989 10.1016/j.jaad.2008.09.056

[4] Sedano HO Ocampo-Acosta F Naranjo-Corona RI Torres-Arellano ME. Multiple dens invaginatus, mulberry molar and conical teeth. Case report and genetic considerations. Med Oral Patol Oral Cir Bucal. 2009;14:E69–72. 19179952

[5] Nissanka-Jayasuriya EH Odell EW Phillips C. Dental stigmata of congenital syphilis: a historic review with present day relevance. Head Neck Pathol. 2016;10:327–31. 26897633 10.1007/s12105-016-0703-z PMC4972761

[6] Hillson S Grigson C Bond S. “Dental defects of congenital syphilis.”. Am J Phys Anthropol. 1998;107:25–40. 9740299 10.1002/(SICI)1096-8644(199809)107:1<25::AID-AJPA3>3.0.CO;2-C

[7] Putkonen T. Dental changes in congenital syphilis. Relationship to other syphilitic stigmata. Acta Derm Venerol. 1962;43:240–9. 14489299

[8] Hutchinson J. Clinical lecture on heredito-syphilitic struma: and on the teeth as a means of diagnosis. Br Med J. 1861;1:515–7. 10.1136/bmj.1.20.515 PMC2287664 20743880

[9] Moon H. On irregular and defective tooth development. Trans Odontol Soc GB. 1877;9:223–43.

[10] Fournier A. Syphilitic teeth. Dent Cosmos. 1884;26:12–25.

[11] Gjestland T. The Oslo study of untreated syphilis: an epidemiologic investigation of the natural course of syphilitic infection based on a restudy of the Boeck-Bruusgaard material. Acta Derm Venereol (Stockh). 1955;35:1–368. 13301322 10.2340/00015555343368

[12] Massler M Schour I. Atlas of the mouth and adjacent parts in health and disease. 1952:16.

[13] Butler PM. Studies of mammalian dentition. – Differentiation of the post-canine dentition. Proc Zool Soc Lond B. 1939;109:1–36.

[14] Dahlberg AA. The changing dentition of man. J Am Dent Assoc. 1945;32:676–90.

[15] Koneru A Hunasgi S Manvikar V Vanishree M. “Nonsyphilitic occurrence of mulberry molars: a rare case report.”. J Oral Maxillofac Pathol. 2019;vol. 23(Suppl 1):106–10. 30967737 10.4103/jomfp.JOMFP_74_18 PMC6421924

[16] Rodrigo C Wijayaratne D Jayawardane M. Hutchinson’s teeth like deformity and aortic dissection in a 31-year-old woman without congenital syphilis. J Symptoms Signs. 2012;1:160–4.

